# Les Bandages, Pansements et Appareils Chirurgicaux

**Published:** 1913-09

**Authors:** 


**Les Bandages, Pansements et Appareils chirurgicaux.** Par Dr. CHARLES JULLIARD. Pp. xii. 221. Geneva: Georg et Cie. 1913. Price 10 fr.

This somewhat elaborate treatise contains a good deal more than is suggested by its title. The book is divided into four sections, dealing respectively with Dressings, Bandages, Extension Apparatus and Splints, the latter term being used in its most comprehensive sense.

A vast amount of information is contained in its two hundred and twenty large magazine pages, and the book should prove useful as a work of reference to anyone wishing to refresh his knowledge of the technique of any method with which he is not familiar.

To quote one example of the comprehensive nature of the contents, an illustrated description is given of the method (attributed to Steinmann, of Berne, and now somewhat fashionable) of applying extension in cases of fracture of the long bones, by means of a pin transfixing the bone below the site of injury.

The book is well printed on good paper, and the illustrations

(over three hundred in number, and mostly from photographs) are excellent, but the binding is flimsy in the extreme. An alphabetically-arranged index would be an advantage.

